# The Association Between Online Social Network Support and Fear of COVID-19 Among Older Adults

**DOI:** 10.1093/geroni/igab046.1141

**Published:** 2021-12-17

**Authors:** Andrew Steward, Matthew Schilz, Kaipeng Wang, M Pilar Ingle, Carson De Fries, Leslie Hasche

**Affiliations:** 1 University of Denver, Lone Tree, Colorado, United States; 2 University of Denver, Denver, Colorado, United States

## Abstract

Public health concerns related to the COVID-19 health crisis are particularly salient among older adults. Fear surrounding COVID-19 has also been associated with increased spread, morbidity, and mortality of the disease. Prior to the pandemic, loneliness and social isolation were already a concern for older adults, and the pandemic further constrained how older adults may socially connect with others because of public health safety precautions. Social networks are a valuable form of support for older adults, and to our knowledge limited prior research has addressed whether social networks are associated with fear of COVID-19. Thus, the purpose of this study is to examine the association between social network and fear of COVID-19 among older adults. A convenience sample (n = 239) of adults 60+ years of age in the U.S. completed a 20-minute survey online or by phone. The independent variable was social network support measured by six items which asked about in-person and virtual contacts with members of the same and other generations. The dependent variable was measured by the Fear of COVID-19 scale (seven items). Results of ordinary least squares regression show that increased social network support was significantly associated with decreased fear of COVID-19 (p < 0.05), while holding constant age, sex, race, marital status, education, whether a respondent lives alone, and self-rated health. Findings highlight the importance of social networks for older adults during the COVID-19 crisis. Existing social networks which engage older adults should be expanded, with the knowledge that such efforts may also help reduce fear of COVID-19 for older adults.

